# Two new inflammatory markers related to the CURB-65 score for disease severity in patients with community-acquired pneumonia: The hypersensitive C-reactive protein to albumin ratio and fibrinogen to albumin ratio

**DOI:** 10.1515/biol-2021-0011

**Published:** 2021-01-22

**Authors:** Bing Luo, Minjie Sun, Xingxing Huo, Yun Wang

**Affiliations:** Department of Clinical Laboratory, Anhui No. 2 Provincial People’s Hospital, Hefei, Anhui 230041, China; Department of Operating Room, Anhui No. 2 Provincial People’s Hospital, Hefei, Anhui 230041, China; Department of Scientific Research Center, The Traditional Chinese Medicine Hospital of Anhui province, Hefei, Anhui 230020, China; Department of Hospital Infection Management, Anhui No. 2 Provincial People’s Hospital, 1868 Dangshan Road, Hefei, Anhui 230041, China

**Keywords:** community-acquired pneumonia, hypersensitive C-reactive protein to albumin ratio, fibrinogen to albumin ratio, CURB-65

## Abstract

**Background:**

The objective of this study was to investigate the relationship among hypersensitive C-reactive protein to albumin ratio (CAR), fibrinogen to albumin ratio (FAR), and the CURB-65 score for community-acquired pneumonia (CAP) severity.

**Methods:**

Clinical data and laboratory indicators of 82 patients with CAP and 40 healthy subjects were retrospectively analysed. The relationship among CAR, FAR, and the severity of CAP was then analysed.

**Results:**

CAR and FAR in patients with low-risk CAP were significantly higher than those in the normal control group (*P* < 0.05). CAR and FAR in patients with medium–high-risk CAP were further increased compared with those in patients with low-risk CAP (*P* < 0.05). CAR and FAR were positively correlated with hypersensitive C-reactive protein, neutrophil to lymphocyte ratio (NLR), platelet to lymphocyte ratio (PLR), and CURB-65 scores (*P* < 0.05). In the receiver operating characteristic curve for predicting severe CAP, the area under the curve of combining four biomarkers (CAR + FAR + NLR + PLR) was the largest. CAR was also an independent risk factor for severe CAP (OR = 8.789, 95% CI: 1.543–50.064, *P* = 0.014).

**Conclusions:**

CAR and FAR may be used as the inflammatory markers for CAP severity evaluation.

## 1 Introduction

Without timely and effective treatment, patients with community-acquired pneumonia (CAP) are at an increased risk for severe CAP, which may cause serious complications, significantly affecting patients' the quality of life and even put their lives in danger [1]. Therefore, the ability to evaluate the severity of CAP and provide reasonable treatment is clinically significant. Most commonly, the CAP severity index scoring system has been used to assess whether patients with CAP can be treated as outpatients or inpatients [2], but the CURB-65 scoring system is also an important tool used to predict the severity of CAP [3]. However, these scoring systems require substantial time and effort to collect multiple sets of patient data. Therefore, various biomarkers have been studied to assess the severity of CAP. Presently, the neutrophil to lymphocyte ratio (NLR), platelet to lymphocyte ratio (PLR), hypersensitive C-reactive protein (hs-CRP), and fibrinogen (FIB) are the primary inflammation markers that have been studied in patients with CAP; of those, the NLR has been shown to be an important marker of disease prognosis and risk stratification in patients with CAP [4,5], whereas NLR and PLR have also been shown to be related to the severity of CAP [6]. However, the area under the curve (AUC) of the receiver operating characteristic (ROC) curve of a single indicator that predicted severe CAP was relatively small, and thus the combined evaluation of multiple markers may provide more valuable diagnostic information.

C-reactive protein to albumin ratio (CAR) and fibrinogen to albumin ratio (FAR) as the new inflammatory markers have become useful indicators to predict the systemic inflammatory response status. Specifically, CAR has been widely used to evaluate the post operative survival rate of patients with pancreatic ductal adenocarcinoma, the prognosis of survival and recurrence in patients with oesophageal cancer, the activity of rheumatoid arthritis, and the severity of coronary heart disease [7,8,9,10]. Another new inflammatory marker, FAR, has shown utility in the evaluation of gastrointestinal stromal tumours, prognosis of resectable gastric cancer, and assessment of rheumatoid arthritis activity [9,11,12]. Moreover, both CAR and FAR have been shown to be correlated with the activity level of rheumatoid arthritis [9]. However, the application of CAR and FAR in the assessment of CAP severity has not been reported in the literature.

We assume that CAR and FAR might be two important markers that can be used to evaluate the severity of CAP. Therefore, this study intends to explore whether CAR and FAR are associated with CAP's severity and compare them with other known markers.

## 2 Materials and methods

### 2.1 Subjects

A total of 82 patients with CAP who presented at Anhui No. 2 Provincial People’s Hospital from January 2018 to April 2019 were classified as the CAP group and were enrolled in this retrospective study. All patients with CAP were diagnosed according to the American Thoracic Society/Infectious Disease Society of America's (2019) diagnostic standard [13]. The following patients were excluded from the study: patients who received other treatment for 3 months, patients with CAP complicated by malignant tumours, other chronic diseases, infection or inflammatory diseases, systemic autoimmune diseases, and cardiovascular and metabolic diseases. The normal control group was composed of 40 healthy subjects matched by age and sex in the physical examination centre.


**Informed consent:** Informed consent has been obtained from all individuals included in this study.
**Ethical approval:** The research related to human use has been complied with all the relevant national regulations, institutional policies, and in accordance with the tenets of the Helsinki Declaration and has been approved by the ethics committee of Anhui No. 2 Provincial People’s Hospital (No. 2017-05).

### 2.2 CAP severity index

The CURB-65 score, which is an important tool used to predict the severity of CAP, assigns different scores to patients according to disturbance in consciousness, blood urea nitrogen level, respiratory frequency, blood pressure, and patient age. The CURB-65 score was used for the classification of CAP severity. The total score ranges from 0 to 5: CURB-65 scores greater than or equal to 0 and less than or equal to 1 indicate low risk; scores equal to 2 indicate medium risk; scores greater than or equal to 3 and less than or equal to 5 indicate high risk [3]. The CURB-65 score was used to assess the severity of CAP at the time of admission, after which the patients were divided into two groups based on the CURB-65 score: the low-risk CAP group included 42 patients with CURB-65 scores greater than or equal to 0 and less than or equal to 1; the medium–high-risk CAP group included 40 patients with CURB-65 scores greater than or equal to 2 and less than or equal to 5.

### 2.3 Clinical evaluation and laboratory data

Clinical evaluation and laboratory data of all patients included age, sex, leukocyte count (WBC), neutrophil (N) count, lymphocyte (L) count, platelet (PLT) count, D–dimer (D–D), hypersensitive C-reactive protein (hs-CRP), albumin (ALB), and FIB. All patients fasted overnight for 8–10 h after admission. Whole blood was collected in the morning for testing according to the requirements of various test indicators. All operations were completed within 2 h in strict accordance with the standard operating procedures of the instrument and the requirements specified in the reagent manual (Sysmex automatic coagulation analyser CS-5100, Japan; Sysmex automatic blood cell analyser XN-1001, Japan; Hitachi automatic biochemical analyser 008AS, Japan). CAR, FAR, NLR, and PLR were calculated according to the results of individual items.

### 2.4 Statistical analysis

SPSS21.0 (Version 21.0, Chicago, IL, USA) and MedCalc15.2.2 were used for the statistical analysis, the measurement data were described by the mean (SD), and gender was shown as male (female). The Kolmogorov–Smirnov test was used to verify data normality, the chi-square test was used for counting data, independent sample *t*-test or one-way ANOVA was used for measurement data, and Pearson correlation analysis was used for linear correlation analysis. Spearman correlation was used for rank correlation analysis. The CURB-65 binary model (CURB-65 ≦ 1 and CURB-65 ≧ 2) was used to analyse the relationship between laboratory parameters and CAP severity by logistic regression. The statistical level was set at 0.05.

## 3 Results

### 3.1 Clinical data and laboratory results of study groups

The hospital records of 82 patients with CAP and 40 gender- and age-matched healthy control subjects were assessed. The clinical data and laboratory results of the two groups of subjects are presented in Table 1. Significant differences were observed in 14 parameters between the two groups but not gender (*P* = 0.585) and age (*P* = 0.239). Patients with CAP had elevated levels of WBC, N, NLR, PLR, D–D, hs-CRP, CAR, FIB, and FAR, whereas their levels of L, PLT, and ALB tended to be decreased (Table 1).

**Table 1:** Clinical data and laboratory results of study groups, mean (SD)

Parameters	Control group (*N* = 40)	CAP group (*N* = 82)	*t*/*χ* ^2^	*P*-value
Gender: male (female)	25 (15)	47 (35)	0.299	0.585
Age: year, mean (SD)	75.93 (8.69)	78.00 (9.29)	1.183	0.239
WBC (10^9^ L^−1^)	6.51 (1.69)	8.83 (4.69)	3.988	<0.001
N (10^9^ L^−1^)	3.71 (1.32)	6.99 (4.67)	5.891	<0.001
L (10^9^ L^−1^)	2.24 (0.73)	1.17 (0.64)	−8.272	<0.001
NLR	1.77 (0.73)	8.64 (8.19)	7.539	<0.001
PLT (10^9^ L^−1^)	247.00 (48.67)	189.02 (75.68)	−5.103	<0.001
PLR	120.16 (43.49)	204.01 (129.36)	5.289	<0.001
D–D (mg L^−1^)	0.13 (0.11)	8.96 (15.47)	5.168	<0.001
ALB (g L^−1^)	47.36 (2.88)	34.68 (5.65)	−16.404	<0.001
hs-CRP (mg L^−1^)	1.80 (3.93)	31.92 (21.37)	12.347	<0.001
CAR (mg g^−1^)	0.04 (0.09)	1.01 (0.75)	11.598	<0.001
FIB (mg L^−1^)	2892.75 (891.77)	4064.27 (1497.18)	5.391	<0.001
FAR (mg g^−1^)	61.47 (21.06)	123.89 (60.19)	8.397	<0.001

WBC: leukocytes; N: neutrophils; L: lymphocytes; NLR: neutrophil to lymphocyte ratio; PLT: platelets; PLR: platelet to lymphocyte ratio; D–D: D–dimer; ALB: albumin; hs-CRP: hypersensitive C-reactive protein; CAR: hypersensitive C-reactive protein to albumin ratio; FIB: fibrinogen; FAR: fibrinogen to albumin ratio.

### 3.2 Clinical data and laboratory results of three groups of subjects

The clinical data and laboratory results of 42 patients with low-risk CAP, 40 patients with medium–high-risk CAP, and 40 healthy subjects were evaluated, and the results are presented in Table 2. Significant differences were observed in the results of all parameters among the three groups except sex (*P* = 0.767) and age (*P* = 0.199). The NLR, PLR, D–D, hs-CRP, CAR, FIB, and FAR were all elevated in patients with low-risk CAP and medium–high-risk CAP, whereas PLT, L, and ALB were decreased, and WBC and N were significantly increased in patients with medium–high-risk CAP compared with the normal control group. In addition, the differences in WBC, N, L, NLR, PLR, D–D, hs-CRP, CAR, FAR, and ALB were statistically significant between the low-risk CAP group and medium–high-risk CAP group (*P* < 0.05) (Table 2).

**Table 2:** Clinical data and laboratory results of three groups of subjects, mean (SD)

Parameters	Control group (*N* = 40)	Low-risk CAP group (*N* = 42)	Medium–high-risk CAP group (*N* = 40)	*F*/*χ* ^2^	*P*-value
Gender: male (female)	25 (15)	23 (19)	24 (16)	0.531	0.767
Age: year, mean (SD)	75.93 (8.69)	76.67 (7.97)	79.40 (10.42)	1.099	0.199
WBC (10^9^ L^−1^)	6.51 (1.69)	7.35 (4.10)	10.38 (4.81)^*#^	11.657	<0.001
N (10^9^ L^−1^)	3.71 (1.32)	5.25 (4.12)	8.81 (4.57)^*#^	20.658	<0.001
L (10^9^ L^−1^)	2.24 (0.73)	1.35 (0.54)^*^	0.99 (0.68)^*#^	38.833	<0.001
NLR	1.77 (0.73)	5.25 (6.26)^*^	12.20 (8.52)^*#^	30.162	<0.001
PLT (10^9^ L^−1^)	247.00 (48.67)	196.12 (67.99)^*^	181.58 (83.22)^*^	10.207	<0.001
PLR	120.16 (43.49)	168.56 (85.84)^*^	241.23 (155.69)^*#^	13.387	<0.001
D–D (mg L^−1^)	0.13 (0.11)	3.75 (5.70)^*^	14.43 (20.08)^*#^	15.462	<0.001
ALB (g L^−1^)	47.36 (2.88)	36.16 (4.70)^*^	33.13 (6.19)^*#^	98.535	<0.001
hs-CRP (mg L^−1^)	1.80 (3.93)	22.58 (20.72)^*^	41.74 (17.43)^*#^	63.182	<0.001
CAR (mg g^−1^)	0.04 (0.09)	0.68 (0.65)^*^	1.37 (0.69)^*#^	58.285	<0.001
FIB (mg L^−1^)	2892.75 (891.77)	3741.43 (1431.05)^*^	4403.25 (1507.48)^*^	13.405	<0.001
FAR (mg g^−1^)	61.47 (21.06)	107.23 (49.26)^*^	141.38 (66.02)^*#^	26.690	<0.001

Note: ^*^ *P* < 0.05, when compared with the normal control group; ^**#**^ *P* < 0.05, when compared with low-risk CAP group.

### 3.3 ROC curve of NLR, PLR, CAR, FAR, and combining four biomarkers (CAR + FAR + NLR + PLR) for predicting severity in patients with CAP

**Figure 1:**
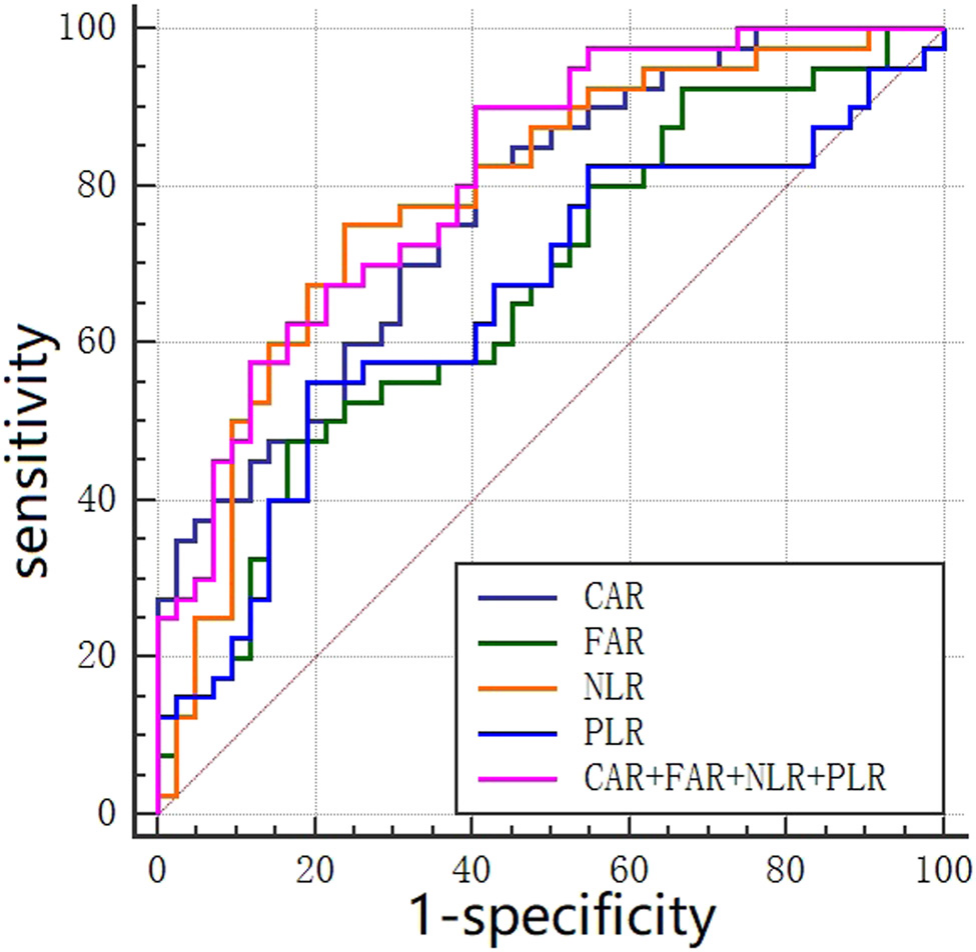
ROC curve of NLR, PLR, CAR, and FAR for severity in patients with CAP.

The prediction efficacy of NLR, PLR, CAR, FAR, and combining four biomarkers (CAR + FAR + NLR + PLR) for severity in patients with CAP was evaluated by comparing the AUC of the ROC curve, which is shown in Figure 1. The results of the AUC analysis are presented in Table 3. The AUC of combining four biomarkers (AUC = 0.813; 95% CI: 0.712–0.891; *P* < 0.001) was the largest, followed by NLR (AUC = 0.786; 95% CI: 0.686–0.887; *P* < 0.001), CAR (AUC = 0.773; 95% CI: 0.675–0.872; *P* < 0.001), FAR (AUC = 0.668; 95% CI: 0.551–0.785; *P* = 0.009), and PLR (AUC = 0.655; 95% CI: 0.535–0.776; *P* = 0.015).

**Table 3:** AUC analysis results of ROC curve of NLR, PLR, CAR, and FAR for predicting severe in patients with CAP

Laboratory indicators	AUC	Standard error	*P*-value	95% CI
NLR	0.786	0.051	<0.001	0.686–0.887
PLR	0.655	0.062	0.015	0.535–0.776
CAR	0.773	0.050	<0.001	0.675–0.872
FAR	0.668	0.060	0.009	0.551–0.785
CAR + FAR + NLR + PLR	0.813	0.046	<0.001	0.712–0.891

### 3.4 Correlation analysis of NLR, PLR, CAR, FAR, and CURB-65 score in patients with CAP

In this study, a bivariate Spearman correlation analysis was performed for NLR, PLR, CAR, FAR, and the CURB-65 score of patients with CAP. As presented in Table 4, the NLR, PLR, CAR, and FAR were positively correlated with the CURB-65 score (*r*_s_ = 0.528, *P* < 0.001; *r*_s_ = 0.271, *P* = 0.014; *r*_s_ = 0.499, *P* < 0.001; *r*_s_ = 0.344, *P* = 0.002, for NLR, PLR, CAR, and FAR, respectively).

**Table 4:** Correlation analysis of NLR, PLR, CAR, FAR, and CURB-65 score in patients with CAP

	NLR	PLR	CAR	FAR
CURB-65	*r*_s_ = 0.528	*r*_s_ = 0.271	*r*_s_ = 0.499	*r*_s_ = 0.344
Score	*P* < 0.001	*P* = 0.014	*P* < 0.001	*P* = 0.002

### 3.5 Correlation analysis of NLR, PLR, CAR, FAR, and other laboratory indicators

**Figure 2:**
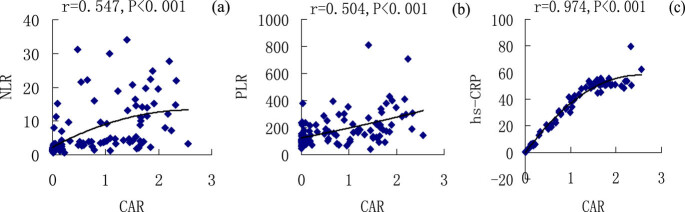
Correlation analysis of CAR with NLR, PLR, and hs-CRP in patients with CAP, CAR, and NLR (a); CAR and PLR (b); and CAR and hs-CRP (c).

**Figure 3:**
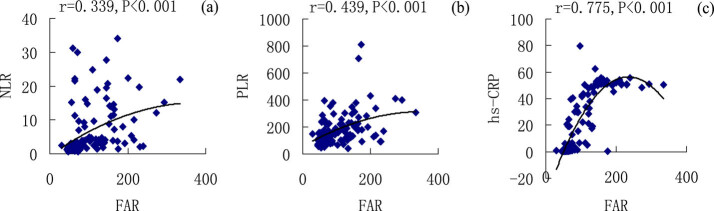
Correlation analysis of FAR with NLR, PLR, and hs-CRP in patients with CAP, FAR, and NLR (a); FAR and PLR (b); and FAR and hs-CRP (c).

In this study, a linear correlation analysis of NLR, PLR, CAR, FAR, and other laboratory indicators was performed using a bivariate Pearson correlation analysis. The results are shown in Table 5 and Figures 2 and 3. CAR was positively correlated with NLR (*r* = 0.547, *P* < 0.001), PLR (*r* = 0.504, *P* < 0.001), and hs-CRP (*r* = 0.974, *P* < 0.001) (Figure 2), whereas FAR was positively correlated with NLR (*r* = 0.399, *P* < 0.001), PLR (*r* = 0.439, *P* < 0.001), and hs-CRP (*r* = 0.775, *P* < 0.001) (Figure 3). NLR was positively correlated with WBC (*r* = 0.676, *P* < 0.001), N (*r* = 0.798, *P* < 0.001), D–D (*r* = 0.311, *P* < 0.001), and FIB (*r* = 0.311, *P* < 0.001) but was negatively correlated with L (*r* = −0.617, *P* < 0.001), PLT (*r* = −0.237, *P* = 0.008), and ALB (*r* = −0.451, *P* < 0.001) (Table 5). PLR was positively correlated with N (*r* = 0.328, *P* < 0.001) and FIB (*r* = 0.343, *P* < 0.001) but was negatively correlated with L (*r* = −0.631, *P* < 0.001) and ALB (*r* = −0.455, *P* < 0.001) (Table 5).

**Table 5:** Correlation analysis of NLR, PLR, CAR, FAR, and other laboratory indicators

Laboratory indicators	CAR		FAR		NLR		PLR	
	*r*	*P*-Value	*r*	*P*-Value	*r*	*P*-Value	*r*	*P*-Value
WBC (10^9^ L^−1^)	0.445	<0.001	0.303	0.001	0.676	<0.001	0.174	0.055
N (10^9^ L^−1^)	0.527	<0.001	0.361	<0.001	0.798	<0.001	0.328	<0.001
L (10^9^ L^−1^)	−0.502	<0.001	−0.378	<0.001	−0.617	<0.001	−0.631	<0.001
PLT (10^9^ L^−1^)	−0.090	0.324	0.019	0.837	−0.237	0.008	0.152	0.095
D–D (mg L^−1^)	0.284	<0.001	0.155	0.089	0.311	<0.001	0.112	0.219
ALB (g L^−1^)	−0.829	<0.001	−0.749	<0.001	−0.451	<0.001	−0.455	<0.001
FIB (mg L^−1^)	0.685	<0.001	0.926	<0.001	0.311	<0.001	0.343	<0.001

### 3.6 Multivariate logistic regression analysis of risk factors for severe CAP

In this study, we used *P* < 0.05 as the critical value to select meaningful variables and eliminate age, sex, FIB, and PLT, and then to select the WBC, N, L, NLR, PLR, D–D, ALB, CAR, and FAR as independent variables to include in the multivariate logistic regression analysis according to the results presented in Table 2. There was a collinearity problem between hs-CRP and CAR with variance inflation factor equal to 12.511, and thus hs-CRP was excluded from the regression model. The updated results are presented in Table 6. Multivariate logistic regression analysis showed that high CAR was an independent risk factor for severe CAP (OR = 8.789, 95% CI: 1.543–50.064, *P* = 0.014).

**Table 6:** Multivariate logistic regression analysis of risk factors for severe CAP

Index	*B*	Wald *χ* ^2^	OR	OR 95% CI	*P*-value
WBC (10^9^ L^−1^)	−1.067	1.188	0.344	0.051–2.343	0.344
N (10^9^ L^−1^)	1.067	1.056	2.906	0.380–22.233	0.304
L (10^9^ L^−1^)	1.538	1.284	4.653	0.326–66.481	0.257
D–D (mg L^−1^)	0.092	3.712	1.096	0.998–1.203	0.054
ALB (g L^−1^)	0.094	0.958	1.098	0.910–1.324	0.328
NLR	0.087	0.778	1.091	0.899–1.324	0.378
PLR	0.000	0.004	1.000	0.993–1.007	0.949
CAR (mg g^−1^)	2.173	5.995	8.789	1.543–50.064	0.014
FAR (mg g^−1^)	−0.004	0.199	0.996	0.979–1.013	0.655

## 4 Discussion

This study's results are as follows: CAR, FAR, NLR, and PLR in patients with CAP were significantly higher than the corresponding values in normal controls. NLR, PLR, D–D, hs-CRP, CAR, FIB, and FAR were higher in patients with low-risk and medium–high-risk CAP compared with those of the normal control group. PLT, L, and ALB were lower than the corresponding values in the normal control group, whereas the WBC and N in patients with medium–high-risk CAP were significantly increased compared with those of the normal control group. Compared with low-risk CAP patients, WBC, N, NLR, PLR, D–D, hs-CRP, CAR, and FAR levels in medium–high-risk CAP patients increased significantly, whereas the levels of ALB and L significantly decreased. In the ROC curve for predicting severe CAP, the AUC of NLR was the largest, followed by CAR, FAR, and PLR. Additionally, CAR and FAR were positively correlated with NLR, PLR, hs-CRP, and the CURB-65 score, but only CAR was an independent risk factor for severe CAP. Therefore, we considered CAR, FAR, NLR, and PLR as important potential predictors of severe CAP.

The severity of CAP depends on the degree of local inflammation, lung inflammation spread, and the extent of systemic inflammation. Studies have shown that neutrophils and platelets are involved in inflammation, which regulates the immune system [14,15], and lymphocytes in patients with CAP are consumed in the anti-infection immunity response [16]. Therefore, NLR and PLR are considered to be representative of the inflammatory status [17,18]. It has been reported that NLR and PLR may be alternative markers of CAP-related inflammation [19,20,21]. Moreover, NLR and PLR have been shown to be predictors of CAP severity [6]. As shown in this study, NLR and PLR are positively correlated with the CURB-65 score and can distinguish the severity of CAP, which is consistent with what is reported in the literature. Therefore, NLR and PLR may be potential predictors of the CAP's severity.

CAR is the ratio of hs-CRP to ALB, and both hs-CRP and ALB are associated with inflammation. hs-CRP belongs to the acute phase reaction protein response, which is increased in inflammatory conditions [22]. At the same time, inflammation can reduce plasma albumin synthesis, resulting in hypoalbuminaemia [9], and thus CAR is a more sensitive predictor of inflammatory status. CAR has been widely used in the evaluation of the post operative survival rate of pancreatic ductal adenocarcinoma patients, the prognosis of colorectal cancer patients after surgery, the activity of rheumatoid arthritis, and coronary heart disease severity [7,8,9,10]. However, thus far, nothing has been published on the relationship between CAR and the severity of CAP. To the best of our knowledge, this is the first clinical study that has shown that CAR levels are elevated in patients with CAP and that a significant increase in the levels of CAR is observed in patients with medium–high-risk CAP compared with patients with low-risk CAP. At the same time, CAR was positively correlated with NLR, PLR, hs-CRP, and the CURB-65 score, and CAR was an independent risk factor for severe CAP compared with the NLR and PLR. This study suggests that CAR may be an inflammatory parameter and a potential predictor of CAP severity.

Similar to CAR and FAR (ratio of FIB to ALB), FIB is also involved in the acute phase reaction protein response, which is increased in inflammatory conditions [23], and inflammatory conditions will reduce the serum albumin levels [9]. In addition, FIB and ALB are associated with CAP [24,25], and these findings have aroused our interest in combining FIB and ALB as a new potential indicator and exploring its relationship with the severity of CAP. In this study, we found a significant increase in the FAR in patients with CAP, whereas the FAR in patients with medium–high-risk CAP was further increased compared with patients with low-risk CAP. However, no statistically significant difference was found in FIB between the low- and medium–high-risk CAP group. Therefore, FAR is superior to FIB in predicting the severity of CAP, and FAR was positively correlated with NLR, PLR, hs-CRP, and the CURB-65 score; therefore, FAR may be an important marker that can be used to assess the severity of CAP.

This study has several limitations that should be discussed. The study's first constraint is a limited retrospective case-control study with relatively small sample size and possible selection bias. The second limitation is that other inflammatory markers related to CAP, such as procalcitonin and interleukins, were not evaluated. The third limitation is that we did not evaluate the effect of treatment on the FAR, CAR, NLR, and PLR.

In conclusion, the development of novel biomarkers and the joint assessment of the severity of CAP are urgently needed and will provide more diagnostic information. To the best of our knowledge, this is the first clinical study to explore the relationship between CAR, FAR, and the severity of CAP. A significant increase in CAR and FAR was observed in patients with CAP, compared with low-risk CAP patients, and CAR and FAR in patients with medium–high-risk CAP were further increased. Additionally, CAR and FAR were positively correlated with the severity of the CAP CURB-65 score. As a result, the CAR and FAR might be two new biomarkers for the evaluation of CAP severity, and because their detection is fast and inexpensive, and they both are easy to analyze, they are worthy of clinical application.
